# Frequency of Left Bundle Branch Block in Patients with Acute Myocardial Infarction; A Cross-Sectional Study

**DOI:** 10.31661/gmj.v8i0.1576

**Published:** 2019-09-02

**Authors:** Reyhaneh Niknam, Mahmonir Mohammadi

**Affiliations:** ^1^School of Medicine, Tehran Medical Sciences Branch, Islamic Azad University, Tehran, Iran; ^2^Department of Cardiology, Faculty of Medicine, Tehran Medical Sciences, Islamic Azad University, Tehran, Iran

**Keywords:** Myocardial Infarction, Acute Myocardial Infarction, LBBB, RBBB

## Abstract

**Background:**

Cardiovascular disease (CVDs) is important problems in both developing and developed countries. Currently, non-invasive methods for diagnosis of CVD, especially myocardial infarction (MI), is an interesting subject in the cardiology field. Some evidence showed left bundle branch block (LBBB) is more prevalent among patients with MI. Hence, this study aimed to investigate the frequency of LBBB and their contributing factors in patients with MI.

**Materials and Methods:**

In this cross-sectional study, 150 patients with ST elevation or non-ST elevation on their admission electrocardiography who referred to Boo-Ali and Amir-Al-Momenin hospitals, Tehran from January 2016 to June 2017 entered the study. Frequency of LBBB and right bundle branch block (RBBB) in participants and the contributing factors were determined.

**Results:**

In this study, of 150 cases (mean age: 60.35±12.88 years), 109 (72.7%) were male, and 41 (27.3%) were female. Out of 150 cases, 12 (8%) had LBBB, 5 (3.3%) RBBB, and 133 (89.7%) had not RBBB or LBBB. Contributing factors were family history, hypertension, and history of ischemic heart disease (P<0.05).

**Conclusion:**

Eight percent of patients with myocardial infarction would develop LBBB, which is related to hypertension, and self and family history of ischemic heart disease.

## Introduction

The likelihood of having obstructive coronary artery disease (CAD) is higher in patients with left bundle branch block (LBBB) [1]. In comparison with normal individuals, patients with LBBB showed significantly higher mortality in the Framingham heart study [1]. Also, the presence of LBBB was found to be associated with an increased risk of progressive heart failure, acute myocardial infarction, and complete atrioventricular block [2]. Patients with CAD tend to have a worse prognosis when associated with LBBB [3]. Diagnose of CAD in patients with LBBB represents a clinical challenge. Noninvasive evaluation of CAD in these patients has several limitations. The available modalities include exercise electrocardiography (ECG), stress echocardiography, and myocardial perfusion imaging, which all become less accurate in the presence of LBBB [4]. One of the challenging diagnostic and therapeutic clinical issues is a patient with suspected acute myocardial infarction (MI) together with LBBB [5]. LBBB and right bundle branch block (RBBB) are diagnosed commonly in routine ECG testing. RBBB often occurs in young patients without apparent organic heart disease, and LBBB more often occurs in older patients with coexisting evidence of organic heart disease and systemic hypertension [6-8]. Algorithms of treatment for patients with acute MI, in accordance with the current European and American guidelines, are based on ECG findings on presentation with ST elevation and non-ST elevation [9]. However, patients with new or presumed new LBBB are a prime example, which does not fall into either of these two categories. Based on the current European guidelines, patients with clinical suspicion of ongoing myocardial ischemia and new or presumed new LBBB should be treated similarly to those with ST elevation MI [5]. The aim of the current study was to investigate the frequency of LBBB in patients with MI.

## Materials and Methods

### Patients

In this cross-sectional study, 150 patients with a definitive diagnosis of acute MI, with ST elevation or non-ST elevation on their admission ECG, referred to Boo-Ali and Amir-Al-Momenin hospitals, Tehran, from January 2016 to June 2017 entered the study (by census sampling method). An expert cardiologist diagnosed acute MI. A final diagnosis of MI also required documentation of two times abnormal troponin levels (troponin>100).

### Sample Size Calculation

The sample size formula was as follows:

$$n=\frac{Z_{\alpha /2}^{2}p(1-p)}{d^{2}}$$

where α=0.05, d=0.01, Z=1.96, and P=0.05

### Data Collection

Information of patients was extracted from their medical history records by a checklist and considering some variables such as age, gender, smoking, and their medical history. Dyslipidemia (total cholesterol>200 mg/dL) and triglyceride level (>150 mg/dL), arterial hypertension (systolic blood pressure/diastolic blood pressure>140/90 mm/Hg) and diabetes (two times fast blood sugar>126mg/dL) were considered if the patients were previously treated for such a condition and/or diagnosed by a physician. Echocardiogram was performed to evaluate heart ejection fraction (EF). Acute anterolateral MI was recognized by ST-segment elevation in leads I, aVL, and the precordial leads overlying the anterior and lateral surfaces of the heart (V3-V6). In a patient with a suspected posterior MI, the endocardial surface of the posterior wall faces the precordial leads, and changes resulting from the infarction would be reversed on the ECG. Therefore, ST-segments in leads overlying the posterior region of the heart (V1 and V2) are initially horizontally depressed. As the infarction evolves, lead V1 demonstrates an R wave–represents a Q wave in reverse. Leads II, III, and aVF reflect ECG changes associated with acute infarction of the inferior aspect of the heart. Currently, we have conventional criteria to diagnose LBBB, including QRS duration>120 msec, QS or RS in lead V1, monophasic R wave with no Q wave in leads V6 and I3, ACC/AHA/HRS added notched R wave in leads I, aVL, V5 and V6 and occasional RS pattern in V5 and V6. Two times abnormal troponin levels and age more than 18 years were considered as acute MI (based on clinical and ECG reports). We also excluded patients with incomplete information in their medical history records.

### Ethical Consideration

This study was approved (code:55119) by the Ethics Committee of Islamic Azad University, Tehran Medical Branch, Tehran, Iran. An informed consent was obtained from all patients, and their personal information remained anonymous.

### Statistical Analysis

Statistical analysis was performed using SPSS software version 16 for Windows (SPSS Inc. Chicago, IL). Qualitative data were expressed as frequency and percent. Also, Independent t-test, Chi-square, and Fisher tests were applied for comparison of quantitative variables. P-values <0.05 were considered statistically significant.

## Results

In this study, of 150 cases, 109 (72.7%) were male, and 41 (27.3%) female. The mean age of patients was 60.35±12.88 years. Of 150 subjects participated, 12 (8%) had LBBB (mean age=63.33±11.7 years), 5 (3.3%) RBBB (mean age=52.6±2.6 years) and 133 (89.7%) had not RBBB or LBBB. No significant association was found between age distribution and LBBB. Seven men (6.4%) and five women (12.2%) had LBBB. There was no significant association between gender and LBBB (P>0.05). In this study, the association between the type and location of MI, addiction, and smoking with LBBB was evaluated, but no significant correlation was seen. Sixty-five patients (43.3%) were smoker. Table-1 shows the demographic data of the patients. In-hospital mortality occurred in nine patients (6%) of 150 patients. Arrhythmia was occurred in 33 patients (22%). Hypertension was found in 54 (36%) of the patients, of which 8 cases (14.8%) had LBBB (P=0.021). This association was statistically significant; thus, the frequency of LBBB in patients with hypertension was significantly higher (Table-1). Based on Table-1, 58 patients (38.7%) were known cases of diabetes mellitus, of whom 5 (8.6%) had LBBB. There was no significant association between diabetes mellitus distribution and LBBB in patients with myocardial infarction. Also, 80 (53.3%) versus 70 (46.7%) of patients had a positive history of hyperlipidemia, of whom 7 (8.8%) of patients with hyperlipidemia had LBBB findings. No significant association was seen between hyperlipidemia and left bundle branch block (P>0.05, Table-1). Among the patients, 41 (27.35%) had a positive history of ischemic heart disease (IHD), and 55 (36.7%) had a positive family history. Also, 88 cases (58.7%) had received streptokinase. Among those with a positive history of IHD, 7 cases (17.1%) had LBBB (P=0.012). In subjects with a history of IHD, the frequency of the LBBB was significantly higher. There was a significant association between positive family history and LBBB (P=0.025). However, the association between receiving streptokinase and LBBB was not significant (P=0.053). Total of 150 patients, 5 cases (3.3%) had RBBB versus 145 (96.7%) without it. The mean EF in patients with LBBB was 44.42 ± 14.89, and in patients without LBBB was 47.2 ± 11.7. No significant association was found between the mean EF of patients and the frequency of LBBB (Table-1). The anterior and the inferior MI had the highest frequency so that 51 cases (34%) had anterior and 72 cases (48%) inferior MI, respectively. Also, the lowest incidence (2.7%) was for posterior MI (Table-2). In 28.7% of patients, no side effects including arrhythmia, cardiogenic shock, and pulmonary edema were seen. Forty (26%) and 18 (12%) patients underwent angiography and percutaneous coronary intervention (PCI), respectively.

## Discussion

In the present cross-sectional study of patients admitted with acute MI, the frequency of LBBB was 8%. Also, there was a significant association between hypertension, history of IHD, and positive family history with LBBB. Our study showed that new LBBB is present in a few patients referred with acute MI. In Jain *et al*. study, the prevalence of LBBB was 4% [10]. In Lopes *et al*. study, LBBB was present in 1.7% [11]. Previous studies have reported rates of 0.5% to 6.7% [12-18], while some other studies reported that patients with new or presumed new LBBB represent a minority of those admitted with acute MI, with a prevalence range of 2% to 9% [10, 19-22]. In the study of Cai *et al*. [23] in the United States in 2013, based on the Sgarbossa’s Criteria, it was possible to identify cases suspected to MI with ST elevation together with LBBB, and those with more than three scores in these comprehensive ranking system were considered as positive cases, which indicates the importance of our study. In a cross-sectional study by Liakopoulos *et al*. [24] in Sweden in 2013, 99 patients were examined that revealed that 33% of patients who did not have LBBB and 37% with LBBB had MI, which indicates a statistically significant association. Neeland *et al*. [20] in 2012 highlighted the importance of a definitive diagnosis of LBBB and MI in the treatment decisions, including the use of reperfusion-therapy. In a cross-sectional study conducted by Jain *et al*. [25] in the United States in 2011, 892 patients with MI were examined, and 36 (4%) of them reported to have LBBB, versus 8% in our recent study. In a cross-sectional that conducted in 2011, 5742 patients with MI were examined, of whom 98 (1.7%) had LBBB, accounting for approximately one-fifth of the value found in our study [26]. This indicates the differences in the various settings and highlights the importance of a separate survey in each center. The presence of LBBB can postpone or complicate the diagnosis of acute MI and also new persistent LBBB in patients with AMI may increase short- and long-term mortality [20, 21]. However, patients with LBBB with clinical symptoms triggered by ischemic MI remain a diagnostic and therapeutic challenge. Erne *et al*. [5] and Liakopoulos *et al*. [27] showed that patients with LBBB were significantly older with a greater burden of risk factors and comorbidities. Others also showed that patients with LBBB were older, and a higher prevalence of comorbid conditions were reported than those without LBBB [10, 14, 15, 22, 28]. Sgarbossa *et al*. [13] proposed specific electrocardiographic criteria for the diagnosis of acute MI in the presence of LBBB based on the criteria performance as applied to 131 patients in the GUSTO-1 trial who had acute MI and LBBB in comparison to patients from the Duke database who had LBBB and were clinically stable. According to Sgarbossa *et al*. study, the Sgarbossa ECG criteria were defined for the diagnosis of acute MI in the setting of a known chronic LBBB [13]. However, it seems that medical history and clinical features of patients with ischemic symptoms and new LBBB do not reliably differentiate which patients have an acute MI [16] and do not show the need for PCI or coronary artery bypass grafting. Therefore, the criteria, such as Sgarbossa criteria, are more to be useful in patients with new LBBB for fibrinolysis [14, 29]. In contrast, these criteria might not be important in patients with ischemic symptoms and any new LBBB, who need urgent angiography. Additionally, overemphasizing on these criteria can postpone reperfusion intervention, which is directly related to the outcomes. European guidelines recommend considering reperfusion therapy using emergency coronary angiography considering primary PCI in patients with myocardial ischemia with new or presumed new LBBB [9]. But in our study, there was no statistically significant association between LBBB and streptokinase. Based on some studies, patients with new or presumed new LBBB were less likely to undergo immediate reperfusion strategies [5]. This finding was reported in some other studies [30]. Our study has some limitations. First, we examined only patients with LBBB and/or RBBB based on medical history records. Also, the low-sample size was another limitation. Hence, future cohesive studies with larger sample size are recommended.

## Conclusion

Based on our findings, eight percent of patients with MI have LBBB, and hypertension, family history and previous history of IHD may increase their risk of developing. Finally, patients with acute MI with new LBBB are a high-risk group and must be treated accordingly. More research is needed given the limitations of studies and additional investigation is needed regarding whether patients with suspected MI and LBBB should be routinely advised for urgent cardiac catheterization.

## Conflict of Interest

None.

**Table 1:** Distribution of LBBB Based on Their Contributing Factors

**Variables**		**LBBB-positive** **n(%)**	**LBBB-negative** **n(%)**
**History of LBBB**		12 (8)	138 (92)
**Smoking**		65 (43.3)	85 (56.7)
**Hypertension**		54 (36)	96 (64)
**Diabetes Mellitus**		58 (38.7)	92 (61.3)
**Hyperlipidemia**		80 (53.3)	70 (46.7)
**IHD**		41 (27.3)	109 (72.7)
**Family History**		55 (36.7)	95 (63.3)
**Streptokinase**		88 (58.7)	62 (41.3)
**BMI**	**<19**	1 (16.7)	5 (83.3)
	**19 – 25**	6 (8.2)	67 (91.8)
	**25 – 29**	3 (5.9)	48 (94.1)
	**>29**	2 (10)	18 (90)
**Ejection Fraction, (Mean±SD)**		44.42 ±14.89	47.20 ± 11.7

**LBBB:** Left bundle branch block; **IHD:** Ischemic heart disease; **BMI:** Body mass index

**Table 2:** Distribution of the Type of MI in LBBB Patients

**Types of MI**	** LBBB-positive** **n(%)**	**LBBB-negative** **n(%)**	**Total** **n(%)**
**Anterior**	7 (13.7)	44 (86.3)	51 (34)
**Inferior**	2 (2.8)	70 (97.2)	72 (48)
**Extensive**	2 (11.8)	15 (88.2)	17 (11.3)
**Posterior**	1 (25)	3 (75)	4 (2.7)
**Antroseptal**	0 (0)	6 (100)	6 (4)
**Total**	12 (8)	138 (92)	150 (100)
